# The role of predatory nematodes in managing plant-parasitic nematodes: community dynamics and microbial implications in tropical soils

**DOI:** 10.3389/fpls.2025.1715934

**Published:** 2025-12-11

**Authors:** Richard Raj Singh, Clancy Iyekar

**Affiliations:** 1 agInnovation Research Center, University of Guam, Mangilao, Guam; 2 Laboratory of Soil Science, University of Guam, Mangilao, Guam

**Keywords:** banana, plant-parasitic nematodes, predatory nematodes, soil organic matter, tropical soil, tomato, temporal interactions

## Abstract

Plant-parasitic nematodes (PPNs) pose a significant threat to banana cultivation, particularly in tropical regions like Guam. This study investigates the dynamics of nematode communities associated with banana cultivars, focusing on interactions between predatory nematodes (*Mononchus* spp.) and PPNs (*Meloidogyne* spp.), and their implications for soil health. Our aim was to evaluate the response of cultivars to nematode prevalence while characterizing nematode community structure and soil chemical properties. Soil and root samples were assessed for nematode communities. Controlled pot experiments were also performed in tomatoes to evaluate predator-prey interactions. The results showed no differences in nematode densities among the banana cultivars, indicating uniform susceptibility to PPNs. We identified three predominant PPN genera: *Meloidogyne* (root-knot nematodes), *Helicotylenchus* (spiral nematodes), and Pratylenchus (lesion nematodes), with *Meloidogyne* as the most dominant. Soil samples contained a higher abundance of bacterivores and predatory nematodes when compared to PPNs. Furthermore, soil analysis revealed high organic matter content and elevated carbon and nitrogen levels. Our temporal analysis revealed that the interaction strength between predatory nematodes and plant-parasitic nematodes (PPNs) increased over time, resulting in a significant reduction in gall formation, egg-laying females, and juvenile populations in tomatoes. This relationship was further quantified through interaction strength (I) values derived from the soil analysis of predatory and root-knot nematodes. In conclusion, our findings highlight the potential for integrating predatory nematodes into pest management. By emphasizing the balance between beneficial nematodes and PPNs, this study provides novel insights applicable to sustainable agricultural practices.

## 1 Introduction

Banana (*Musa* spp.) is one of the most important fruit crops cultivated in tropical and subtropical regions, serving as a staple food, cash crop, and crucial source of livelihood for millions globally (FAO, 2021). In Pacific Islands, bananas hold significant cultural and economic importance, contributing not only to local food security but also enhancing agricultural sustainability (Campbell, 2015; Georgeou et al., 2022). The island of Guam cultivates several banana cultivars, including Dwarf Cavendish, Williams Hybrid (Giant Cavendish) and other cavendish group, each varying in agronomic traits such as fruit size, yield potential, disease resistance, and adaptability to different soil conditions (Scully and Bevacqua, 1988; Ara et al., 2012).These variations directly influence their interactions with soil microorganisms, including plant-parasitic nematodes (PPNs) (Gowen and Quénéhervé, 1990; Gaidashova et al., 2008; Sousa et al., 2024).

Nematodes are microscopic roundworms inhabiting diverse soil ecosystems, where they can either benefit or harm plant health. PPNs cause a significant threat to agricultural productivity by not only causing damage individually but forming disease-complexes with other microorganisms and increased crop loss (Singh et al., 2015). These nematodes invade root tissues, causing extensive damage that impairs nutrient and water uptake, ultimately leading to reduced plant vigor, stunted growth, and premature decline (Lopes-Caitar et al., 2019; Özarslandan et al., 2020). Root-knot nematodes (RKN) *Meloidogyne* spp. are particularly destructive due to their capacity to induce gall formation on roots on a wide range of crops, for example in tomato (Mekete et al., 2003; Bouchtaoui et al., 2025; Gupta et al., 2025) in pepper (Mekete et al., 2003) in rice (Kyndt et al., 2012; Nguyễn et al., 2014; Singh et al., 2019, Singh et al., 2020). Other PPNs, such as burrowing nematode *Radopholus* spp. and lesion nematode *Pratylenchus* spp., cause necrosis and lesions that further compromise plant health (Thompson et al., 2008; Kavitha et al., 2025). In banana cultivation, *Meloidogyne* spp, *Radopholus* spp., *Pratylenchus* spp., *Helicotylenchus* spp., *Rotylenchulus* spp. and *Tylenchus* spp. are commonly linked to reduced yields and compromised plant health (Gowen and Quénéhervé, 1990; De Waele and Davide, 1998; Daneel and De Waele, 2017; Sikora et al., 2018). While PPNs pose considerable challenges for crop production, various nematode groups also play beneficial roles within soil ecosystems (Neher, 2001; Pires et al., 2023; Semprucci et al., 2025). Free-living nematodes, including bacterivores, fungivores, and omnivores, contribute to soil nutrient cycling by decomposing organic matter and regulating microbial populations (Li et al., 2024; Martin, 2025). Notably, predatory nematodes, such as *Mononchus* spp., have emerged as potential biological control agents against PPNs by preying on other nematodes, including *Meloidogyne* juveniles, thus reducing their population densities and limiting infection rates in host plants (Khan and Kim, 2007; Kim, 2015; Shokoohi, 2024; Martin, 2025). The diverse soils of southern Guam, including volcanic-derived clay soils and limestone-derived sandy loams, present a unique environment for crop cultivation. These soils exhibit varying physical and chemical properties that influence their ability to support healthy banana production. Volcanic clay soils, which typically possess high water-holding capacity, can become prone to compaction, potentially limiting root growth and aeration (Scully and Bevacqua, 1988; Miller et al., 2002). Conversely, limestone soils, found in coastal regions, are well-drained but often exhibit low organic matter content, making them vulnerable to nutrient leaching and degradation (Stanturf and Schoenholtz, 2019; Gerke, 2022). To enhance soil quality and support sustainable agriculture, soil amendments such as compost and soil organic matter incorporation are essential (Diacono and Montemurro, 2011; Matisic et al., 2024). The addition of organic amendments can significantly increase soil fertility, improve soil structure, and boost microbial activity (Diacono and Montemurro, 2011; Aytenew and Bore, 2020; Zhou et al., 2025). Moreover, such practices promote the proliferation of beneficial organisms, including predatory nematodes, which can help mitigate the effects of PPNs (Akhtar and Malik, 2000; Oka et al., 2007; Thoden et al., 2011; Malik et al., 2025). Research shows that compost applications can stimulate microbial antagonists that suppress nematode populations, thereby reinforcing the use of organic inputs as a strategy in integrated pest management (Zhen et al., 2014; Tóthné Bogdányi et al., 2021; Karanastasi et al., 2024).

In this study, we aimed to investigate the response of different banana cultivars to nematode prevalence in Guam’s tropical soil condition while characterizing the nematode community structure and trophic group dynamics. Additionally, the interactions between predatory nematodes (*Mononchus* spp.) and RKN (*Meloidogyne* spp.) were examined through a controlled pot experiment using tomato plants as a model system. This multifaceted approach provides valuable insights into the biological control potential of predatory nematodes and their ecological implications for nematode populations in tropical agricultural systems.

## 2 Materials and methods

### 2.1 Study site and experimental design

This study was conducted at the Inalåhan Research & Education Center (GPS coordinates: 13°16′32″N, 144°44′36″E) in the southern region of Guam, where soil fertility is optimal for banana cultivation.(**Supplementary Figure S1**). Five banana cultivars (Fiji, Blue Java, Macau, Manila, and Saba) were evaluated in a completely randomized block design, with seven replications per cultivar. Experimental units consisted of single plants, allowing for precise control and assessment of nematode interactions. The total area covered by the banana trial was 4,800 sq ft (80’ x 60’). Sampling was conducted in October 2024 and again in January 2025.

### 2.2 Nematode analysis

#### 2.2.1 Soil and root sampling

Soil and root samples were systematically collected to assess nematode communities. Sampling was performed with certain conditions as instructed in Ferris et al. (1981). To accurately assess soil health, nematode populations, and overall plant-soil interactions, we performed “root-zone sampling,” specifically focusing on the soil adjacent to banana plant roots. A total of 3 soil samples were taken per mat for each replicate. Given that there were 7 replicates for each banana cultivar, this resulted in 21 individual samples per cultivar. To create composite samples for analysis, we combined the 3 samples from each replicate into a single composite sample. Thus, we ultimately analyzed 7 composite samples for each banana cultivar. This rhizosphere soil, enriched with microorganisms, nutrients, and organic matter, provides a dynamic environment where nematode groups thrive based on their feeding activities. Soil was sampled from the rhizosphere at a depth of 15 cm using a soil auger. Feeder roots were harvested with a clean knife. For root sampling, we collected 3 samples per plant from each banana cultivar. A total of 7 plants were sampled per cultivar, resulting in a comprehensive assessment of the root system for each cultivar (Arieira et al., 2016). Samples were transferred to lab for further processing.

#### 2.2.2 Nematode extraction from soil rhizosphere

For nematode extraction, 100g bulk soil sample from the rhizosphere was used, employing the Baermann funnel method (Baermann, 1917) with minor modifications. Initially, soil samples were sieved through a 2 mm mesh to eliminate larger debris. The fine soil fractions were then placed in a funnel apparatus set over a container of water and incubated at 25 °C. Due to their sensitivity to light and heat, nematodes migrate from the soil into the water below. After 48 hours of extraction setup, nematodes were collected in a volume of 5–10 mL of water (Baermann, 1917; Hallmann and Subbotin, 2018). The composition of soil nematofauna was analyzed by fixing samples in a formaldehyde glycerol solution and placing them on permanent slides.

#### 2.2.3 Nematode extraction from banana roots

The nematodes were recovered from the root tissue using protocol as instructed in (Coolen and D’herde, 1972; Speijer and Ssango, 1999; Hooper et al., 2005). Five root pieces were cut into 1-cm segments and combined. A 5-g subsample was added to 50 ml of water and blended at medium speed for 10 seconds. The resulting mixture was poured onto two layers of Bounty paper toweling suspended in a Baermann tray, ensuring the water level touched the roots (McSorley, 1987). After two days, the water was replaced with fresh water to boost extraction efficiency (Brooks, 2004), likely by increasing the water’s oxygen content. A final count of nematodes was conducted at five days, combined with the initial two-day count, and adjusted to a density of 100 g fresh root weight.

#### 2.2.4 Morphological and morphometric analysis

Extracted nematodes were fixed with a hot 4% formaldehyde solution, preserved in an anhydrous glycerin utilizing the procedure described in Coyne (2007) and mounted on microscopic glass slides, examined using a compound microscope, identifying species based on morphological characteristics such as body shape, size, tail morphology, and presence of a stylet (Yeates et al., 1993; Spiegel, 2005; Coyne, 2007; Yeates, 2009). Morphometric measurements, including body length, width, and tail shape, were recorded and matched against published descriptions Cobb (1890); Kaur and Attri (2013); Jesus et al. (2020) to confirm species identification.

#### 2.2.5 Determination of nematode density in roots and soil

To evaluate the nematode density, we performed manual counting method with subsampling (Drop method) as instructed in Barker and Noe (1988); Coyne (2007); Shokoohi (2025). Nematode suspension was collected and well-mixed to distribute the nematodes evenly. A micropipette was used to transfer a small, fixed volume (1mL) of the suspension to a counting dish with grid patterns. The nematodes were counted under a dissecting microscope. The process was repeated with multiple aliquots (5) and the average count was determined and nematode was presented as 100 cm^3^ of soil or 100g of roots.

#### 2.2.6 Nematode community analysis

Nematode community structures were assessed by calculating the relative abundances of functional groups (bacterivores, fungivores, predatory, and plant-parasitic nematodes) using documented methods in Ferris et al. (2001). This assessment provides insight into the ecological dynamics within different soil types and the potential impact of soil amendments.

### 2.3 Soil chemical analysis

#### 2.3.1 Measurement of pH

To determine the pH of soil samples, we utilized a one-to-one ratio of soil to distilled water. Specifically, 10 grams of the soil sample was weighed and transferred to a glass beaker. 10 milliliters of distilled water were added to the sample, and the mixture was stirred for 30 seconds to ensure thorough mixing. The suspension was allowed to sit undisturbed for 10 minutes, after which the pH was measured using a calibrated PHS-3DW Benchtop pH Meter (DX-PHS-3DW, VWR International).

#### 2.3.2 Organic matter analysis

The organic matter content of the soil was analyzed using the Walkley & Black method, a titration process based on potassium dichromate and iron sulfate. About 0.2 grams of soil sample was weighed into a 250 mL Erlenmeyer flask and 10 milliliters of 1N potassium dichromate were added, followed by 10 milliliters of concentrated sulfuric acid. The mixture was allowed to digest for 30 minutes at room temperature. After digestion, five drops of 0.025M phenanthroline ferrous complex were added to the flask. Subsequently, 100 milliliters of distilled water was added, and the sample was titrated with 0.5N ferrous sulfate until the endpoint was reached, which was indicated by a color change. A blank was prepared and treated in the same manner to determine the percent organic matter content, with results compared against soil organic matter data from Guam soils (**Supplementary Table S1**).

#### 2.3.3 Carbon and nitrogen analysis

Soil samples were finely ground to a powdery consistency to facilitate accurate measurement of carbon and nitrogen content. About 10 micrograms of the powdered sample were weighed and analyzed using a Soil Carbon/Nitrogen Analyzer (Flash EA 1112, Manufacturer). The samples were ignited in the analyzer, converting carbon and nitrogen into gaseous forms. The emitted gases were filtered and scrubbed to isolate carbon and nitrogen, which were then detected by the analyzer’s thermal coupled detector, providing total percentages of carbon and nitrogen. The results were compared against % Carbon and % Nitrogen data from Guam soils (**Supplementary Table S1**).

### 2.4 Predator–prey relationships analysis

#### 2.4.1 Plant preparation

Tomato seeds (*Solanum lycopersicum* cv. Moneymaker) were germinated in sterilized soil under controlled conditions, maintained at 25 ± 2 °C, with 70% relative humidity and a 16/8-hour light/dark photoperiod. The seedlings were allowed to establish for three weeks to develop robust root systems before transplanting. During the transplanting process, care was taken to minimize root damage and stress to the seedlings, ensuring optimal conditions for nematode colonization (**Supplementary Figure S2A**).

#### 2.4.2 Pure nematode populations

Populations of the predatory nematode *Mononchus* sp. and RKN *Meloidogyne incognita* were utilized to assess the dynamics of the predator-prey relationship. The initial stock of *M. incognita* was obtained from pure populations raised in tomato plants (*Solanum lycopersicum* cv. Moneymaker) under glasshouse conditions at 23 °C with a 16:8-hour light/dark cycle in Guam. The identification of *M. incognita* was confirmed using morphological features observed through microscopic examination. Key characteristics included the anterior part of the female, which displayed a visible stylet and knob, essential traits for identifying root-knot nematodes. Additionally, the second-stage juvenile (J2) was characterized by its distinct head structure and tail region, which are critical for accurate species identification (**Supplementary Figure S2B**). Infected plants were carefully harvested, and galls with egg masses were collected from the roots, which were then allowed to hatch in water. Freshly harvested second-stage juveniles (J2) were used as inoculum in the predator/prey infection experiments.

The predatory nematode *Mononchus* sp was raised in sterilized sand–soil mixtures supplemented with organic litter as a substrate for microbial colonization and proliferation of bacterivores nematodes as instructed in Salinas and Kotcon (2005); Bilgrami (2008). A starter population of bacterivores nematodes (mainly *C. elegans*) was introduced as prey. *C. elegans* was cultured in bacterial prepared culture plates bought from Carolina Biological supply. Cultures were maintained at 25 ± 2 °C and kept moist with sterile distilled water. *Mononchus* populations were collected by Baermann funnel extraction and used as inoculum.

#### 2.4.3 Evaluation of infection and interaction strength

To evaluate the interaction between the predatory nematode *Mononchus* and the plant-parasitic nematode *Meloidogyne incognita*, infection experiments were established using five-week-old tomato seedlings. Plants were grown in growth medium consisted of sterilized soil that mimicked the high organic matter (OM) and carbon (C) content found in the field. Tomato seedlings (*S. lycopersicum*), 5 weeks old, were inoculated with *Meloidogyn*e juveniles (RKN) followed by co-inoculation of predatory (RKN + P). Control pots received no predators (RKN). The plants were inoculated with 250 RKN solo or co-inoculated with 250 predatory by distributing the inoculum into two holes (ca. 2.5-cm deep) at the base of the plant as instructed in Timper et al. (2016). The roots were then assessed for gall formation, egg laying females and juvenile stages presence at 4-, 6- and 8-weeks post inoculation. The timeline depicting the age of tomato seedlings and the corresponding inoculation and assessment schedule are simplified in (**Supplementary Figure S3**) All assessments (galls, egg-laying females, and juveniles) originated from the same plant. For gall assessment, roots were carefully washed under running tap water, and the number of galls per root system was recorded by visual inspection (Bridge and Page, 1980)). To quantify egg-laying females, galled roots were further washed in 0.5% sodium hypochlorite as instructed in Hussey and Barker (1973) rinsed thoroughly with distilled water, and examined under a stereomicroscope for the presence of egg masses, with brown egg masses considered indicative of mature, reproducing females (McClure et al., 1973). To evaluate juvenile populations inside roots, samples were stained in acid fuchsin following the protocol of Nahar et al. (2012); Singh et al. (2020) and destained in acidified glycerol (glycerol + 1 ml of 37% HCl) as indicated in Desmedt et al. (2022) and migratory second-stage juveniles (J2) were counted microscopically. Replicates consisted of eight plants for each treatment.

#### 2.4.4 Assessment of predator–prey interaction strength

To quantify the interaction strength between predatory nematodes (*Mononchus* sp.) and PPN (*Meloidogyne incognita*), we assessed the number of parasitic juveniles and predatory nematodes present in a defined volume of soil (100 cm³). This measurement facilitated a standardized approach to comparing the population dynamics of the two nematode types under varying treatment conditions. To further explore these interactions, we included an additional treatment involving predatory nematodes presented as a standalone group. This setup was designed to evaluate the direct effect of predators on prey populations over different temporal intervals. By monitoring changes in both predator and prey populations at multiple time points, we aimed to gain insights into the dynamics of their interactions across different stages of nematode development.

#### 2.4.5 Interaction strength calculation

Interaction strength (I) provides a measure of the impact a predator has on its prey population.

According to Paine (1980; 1992) whereby interaction strength is defined by the formula:


$$I=\left(D_{P}–D_{O}\right)/\left(D_{P}\right)P$$

Where P = the number of predators in the predator (inclusion) treatment and,

D_O_ = the number of preys remaining after a specified amount of time in the predator exclusion treatment (no predator present, so only natural mortality and methodological problems lead to loss of prey.

D_P =_ the number of preys remaining in the predator inclusion treatment.

Negative values of I indicate a significant top-down effect, illustrating the efficiency of predation on juvenile nematode populations.

### 2.5 Statistical analysis

Data were analyzed using R version 4.1.2, employing a heteroskedastic *t*-test for comparisons between two groups and Tukey’s range test for multiple comparisons after assessing normality and homoscedasticity assumptions through normal Q-Q and residual plots. Different feeding groups were categorized, with the resulting data transformed into relative abundances. Simpson’s diversity index was utilized to estimate alpha diversity using the diversity function within the VEGAN package. The abundance of each genus was compared using the Kruskal-Wallis test, followed by FDR-adjusted pairwise Wilcoxon-Mann-Whitney tests for *post-hoc* comparisons. For tomato pot experiment statistical significance was evaluated using one-way ANOVA, followed by Tukey’s *post-hoc* analysis, with *P*-values indicating levels of significance at *α* = 0.05 for general significance, and values less than 0.001 indicating highly significant differences among treatments. A total of 8 replicates were conducted for each treatment, and the experiment was repeated three times, with data pooled for a comprehensive analysis (n = 8). Error bars in the figures represent the standard error of the mean (SEM), providing insight into data variability.

## 3 Results

### 3.1 Prevalence of plant parasitic nematodes in banana roots

**Figure 1:**
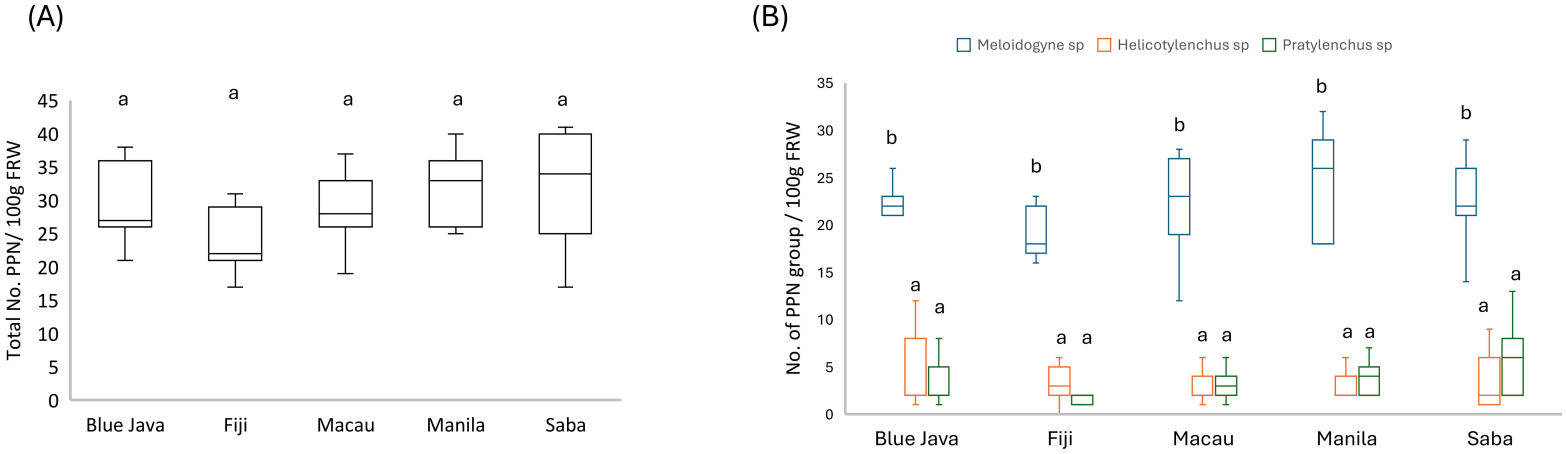
Comparison of plant-parasitic nematode (PPN) populations across banana cultivars. Total number of PPN per 100 g of fresh root weight (FW) among five banana cultivars: Blue Java, Fiji, Macau, Manila, and Saba **(A)**. Number of specific PPN genera per gram in soil groups across the same banana cultivars. Three PPN groups are depicted: *Meloidogyne* spp., *Helicotylenchus* spp., and *Pratylenchus* spp., with their respective distributions indicated by separate box plots **(B)**. Data are represented as box plots, showing the median, quartiles, and range of PPN numbers for each cultivar. A total of 7 replicates were conducted for each measurement, and sampling and analysis were performed twice to ensure reliability. The data were pooled for comprehensive statistical analysis to account for variability across replicates. Different letters showing statistical differences (*p<* 0.05).

To assess the susceptibility of banana cultivars namely Blue Java, Fiji, Macau, Manila, and Saba to nematode infestation, we conducted an analysis of the roots from each cultivar for nematode prevalence. Initially, we examined the total number of PPNs present in the roots of the various cultivars. Our findings indicate that all cultivars displayed comparable levels of susceptibility (**Figure 1A**). The total number of PPNs detected ranged from 22 to 34; although Fiji exhibited a lower count of PPNs, this difference was not significant when compared to the other cultivars (**Figure 1A**). Subsequently, we aimed to identify genera of PPNs commonly associated with the roots of all cultivars. Our results revealed that all banana cultivars were susceptible to three primary genera of PPNs: root-knot nematodes; *Meloidogyne* spp, and migratory endoparasitic nematodes *Helicotylenchus* spp, and *Pratylenchus* spp. *(***Figure 1B**). Notably, the prevalence of *Meloidogyne* sp. was greater (~4-5X higher) than that of *Helicotylenchus* sp. and *Pratylenchus* sp. across all cultivars (**Figure 1B**).

### 3.2 Prevalence of nematodes in the banana root rhizosphere

**Figure 2:**
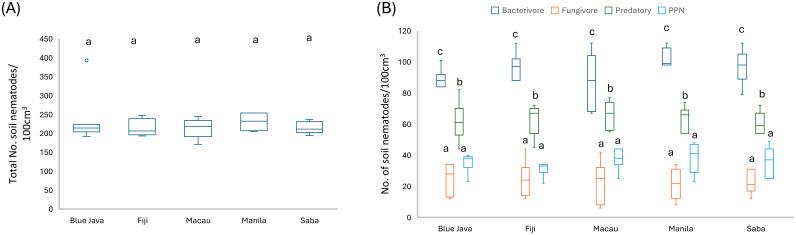
Soil nematode population dynamics across different banana cultivars. Total number of soil nematodes per 100 cm³ of soil for five banana cultivars: Blue Java, Fiji, Macau, Manila, and Saba **(A)**. Distribution of nematode feeding groups per 100 cm³ of soil across the same banana cultivars. The feeding groups include Bacterivores, Fungivores, Predatory nematodes, and Plant-Parasitic Nematodes (PPN), each represented by distinct colors in the box plots **(B)**. Data are presented as box plots, illustrating the median, quartiles, and range of nematode numbers. A total of 7 replicates were conducted for each measurement, and sampling and analysis were performed twice to ensure reliability. The data were pooled for comprehensive statistical analysis to account for variability across replicates. Different letters showing statistical differences (*p<* 0.05).

**Figure 3:**
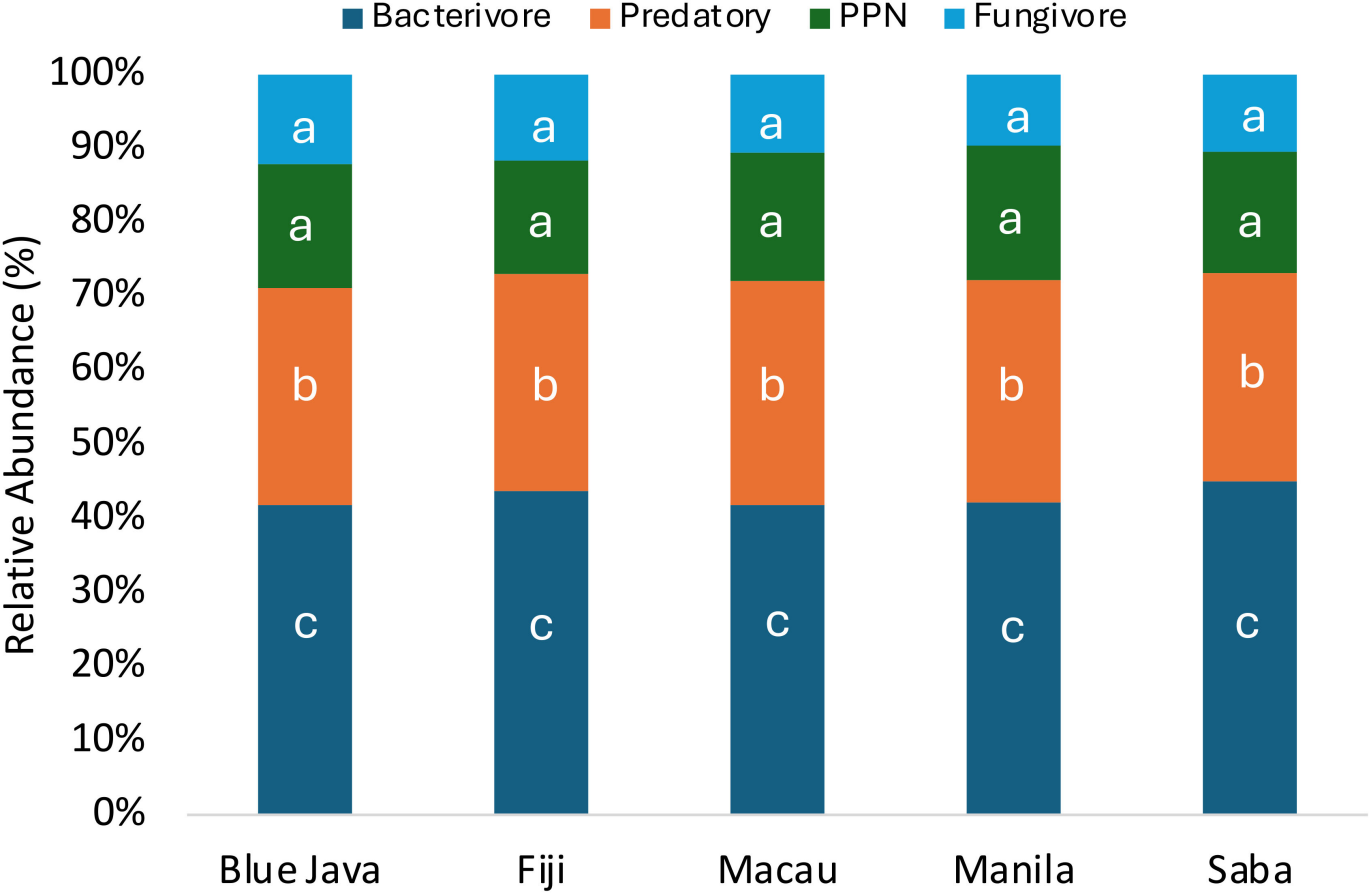
Relative abundance of nematode feeding groups in the root rhizosphere of five banana cultivars. The relative abundance of Bacterivores, Predatory nematodes, Plant-Parasitic Nematodes (PPN), and Fungivores, across five banana cultivars—Blue Java, Fiji, Macau, Manila, and Saba. Each bar represents combined data from two rounds of sampling and analysis, with seven replicates per cultivar. The percentage values indicate the proportion of each feeding group relative to the total nematode population for each cultivar, calculated by dividing the number of nematodes in each feeding group by the overall total and multiplying by 100. Simpson’s diversity index was utilized to estimate alpha diversity using the diversity function within the VEGAN package. The abundance of each genus was compared using the Kruskal-Wallis test, followed by FDR-adjusted pairwise Wilcoxon-Mann-Whitney tests for *post-hoc* comparisons. Different letters showing statistical differences (*p<* 0.05).

To accurately assess soil health, nematode populations, and overall plant-soil interactions, we performed “root-zone sampling,” specifically focusing on the soil adjacent to banana plant roots. Our findings revealed different nematode feeding groups: bacterivores, fungivores, predatory nematodes, and PPNs present in the rhizosphere samples of all cultivars. The total number of soil nematodes was evaluated across the root rhizosphere of five banana cultivars. Our results revealed no significant differences in the total number of soil nematodes among the different cultivars (**Figure 2A**). Contrastingly, the most dominant group was the bacterivores nematodes, which were present in significantly higher numbers compared to all other feeding groups (**Figure 2B**). Surprisingly, the next most prevalent group were the predatory nematodes, which was also significant higher (2x higher) when compared to PPNs and fungivores nematodes (**Figure 2B**). To further complement our initial findings, we conducted an analysis of the relative abundance for each feeding group to understand their distribution and dominance. Our studies shows that there is a uniform distribution of these feeding groups across all cultivars (**Figure 3**). Our data shows that bacterivores nematodes constitute approximately 40% of the total nematode population, being the most abundant across all cultivars while predatory nematode on the other hand consist of up to 30% of the nematode community, fungivores occupied about 17% of the nematode assemblage and with a relative abundance of 13%, plant-parasitic nematodes represented the smallest portion of the community (**Figure 3**).

### 3.3 Soil analysis and nematode prevalence in the root rhizosphere

Given the significant presence of bacterivores and predatory nematodes along with correspondingly lower number of PPNs in the banana root rhizosphere (**Figures 2B**, **3**), we conducted a comprehensive soil analysis to evaluate the soil pH and organic matter content, as well as the percentages of nitrogen and carbon in the soil, as these factors may influence nematode prevalence. Conversely, the activity of nematodes and other microorganisms can also affect soil properties such as organic matter and nutrient levels. Our analysis considered key soil properties across plots of the five banana cultivars: Blue Java, Fiji, Macau, Manila, and Saba. Our data shows no significant differences across the cultivars for several key soil parameters (**Table 1**). The pH values across all plots were relatively consistent, ranging from a mean of 6.54 in the Blue Java cultivar to 7.13 in Fiji, indicating slightly acidic to neutral conditions conducive to nematode activity. The organic matter content showed minimal variation between the cultivars. The mean organic matter ranged from 8.17 in Saba to 8.75% in Fiji. Furthermore, no significant differences were observed in the % total carbon and % total nitrogen content in the root rhizosphere sample across different cultivars. To contextualize our findings, the results obtained for pH, organic matter, carbon, and nitrogen content were compared with established soil data from Guam soil analysis reference data for the region (**Supplementary Table S1**). This comparison was crucial for evaluating the nutritional and health status of the study soils relative to typical profiles found in Guam soils.

**Table 1:** Soil chemical properties across different banana cultivars.

pH				% O.M.			Total % C			Total % N		
Cultivar	Mean	SE	Sig level	Mean	SE	Sig level	Mean	SE	Sig level	Mean	SE	Sig level
Blue Java	6.54	0.05	a	8.55	0.53	a	4.1	0.1	a	0.26	0.00	a
Saba	6.78	0.03	a	8.17	0.32	a	4.7	0.2	a	0.36	0.01	a
Macao	6.98	0.01	a	8.21	0.2	a	4.4	0.1	a	0.32	0.00	a
Manilla	6.96	0.01	a	7.98	0.3	a	3.8	0.2	a	0.32	0.00	a
Fiji	7.13	0.03	a	8.75	0.48	a	4.2	0.4	a	0.31	0.01	a

This table presents the mean values, standard errors (SE), and significance levels for soil chemical properties measured around five banana cultivars: Blue Java, Saba, Macau, Manila, and Fiji. The properties analyzed include soil pH, percentage of soil organic matter (% S.O.M.), total soil carbon (Total % C), and total soil nitrogen (Total % N). Different letters showing statistical differences (*p<* 0.05).

### 3.4 Temporal effects of co-inoculation with *Mononchus* spp. and *Meloidogyne* spp.

**Figure 4:**
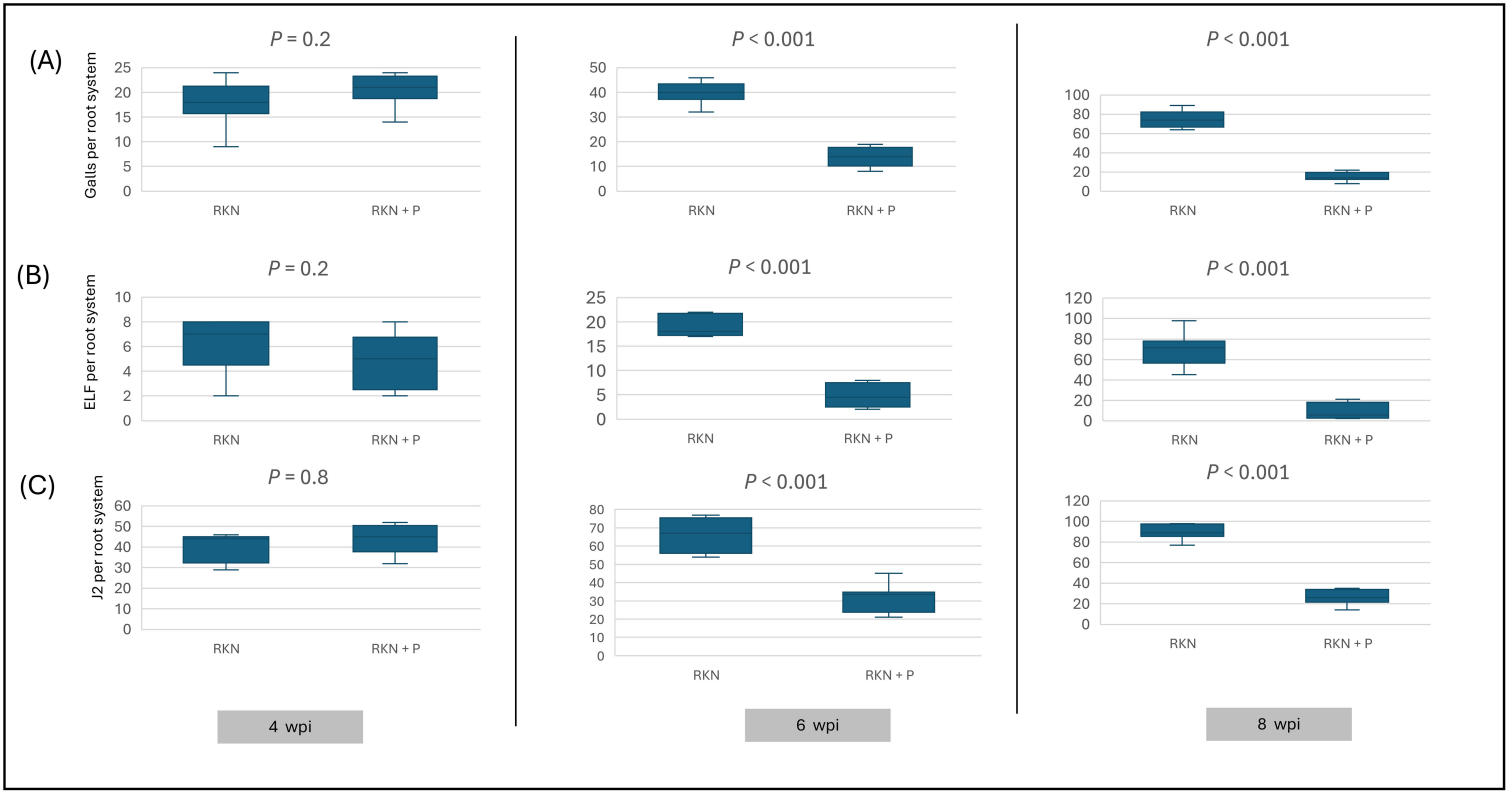
Temporal dynamics of root-knot nematode populations in banana cultivars with and without predatory nematode introduction. The number of galls per root system observed in tomato pots **(A)**. The number of egg laying females (ELF) observed in tomato pots **(B)**. The number of juveniles per root system observed in tomato pots **(C)**. The pots were co-inoculated with RKN and predatory nematodes (RKN + P) and control pots were observed at 4, 6, and 8 wpi for each of the parameter. Data are presented as box plots, showing median values, interquartile ranges, and minima/maxima. Statistical significance was evaluated using ANOVA followed by Tukey’s *post-hoc* analysis, with *p*-values indicating the level of significance. A total of 8 replicates were used for each treatment, and the experiment was repeated three times, with the data pooled for comprehensive analysis.

**Figure 5:**
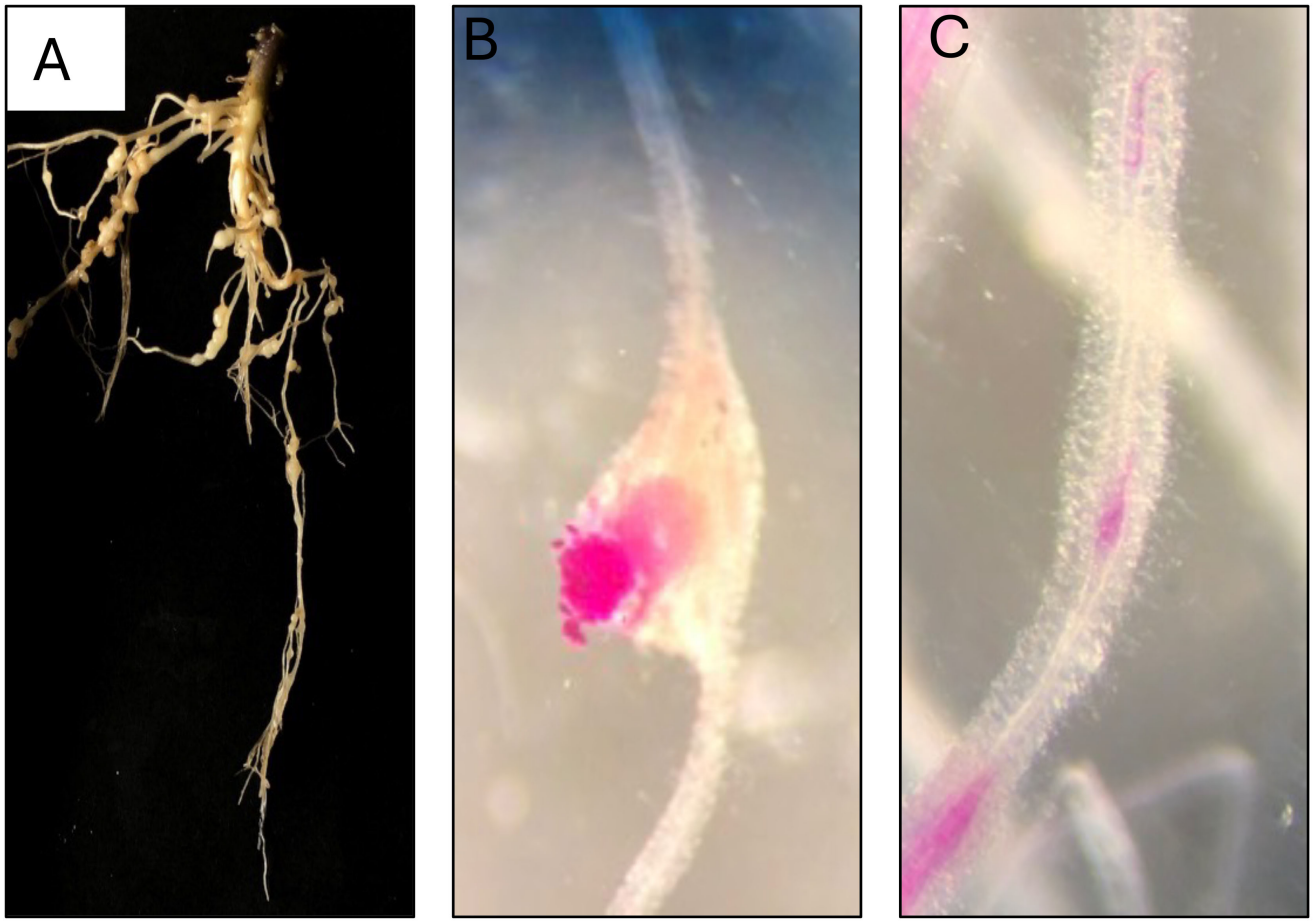
Root-Knot Nematode (*Meloidogyne* spp.) infection in tomato plants. *Meloidogyne*-induced galls with the characteristic of galls, or swellings, that form on the roots of tomato plants (*Solanum lycopersicum*) as a result of root-knot nematode (RKN) infection **(A)**. Egg-laying females is depicted on the surfaces of the galls **(B)**. The juvenile nematode stages present within the roots of the tomato plants **(C)**.

Given the observed differences in the prevalence of predatory nematodes and PPNs in the field, we conducted a follow-up pot experiment to investigate predator-parasite interactions under controlled laboratory conditions. The high number of predatory nematodes and the low number of PPNs led us to hypothesize that the predatory nematodes may be feeding on PPNs, which could explain the reduced populations of the latter. Three independent experiments were conducted to assess the impact of predatory nematodes on PPNs: root knot nematode (RKN) over 3 different time points at 4-, 6, and 8-weeks post-inoculation (wpi). Model crop, tomato seedlings were inoculated with 250 RKN *Meloidogyne* juveniles and co-inoculated with 250 predatory nematodes (RKN + P). Control pots received no predators (RKN). Our experiment first investigated the effects on RKN-induced gall formation (**Figures 4A**, **5A**), as galls serve as crucial feeding sites for RKN. Our data shows no significant difference in the number of galls between the RKN (control) and the RKN co-inoculated with predatory nematode (RKN + P) treatment (*p = 0.2*) at 4 weeks (**Figure 4A**). At weeks 6 and 8, a significant reduction (3 & 5x reduction respectively) was observed in gall numbers in the RKN + P treatment when compared to control (*p< 0.001).* Furthermore, to assess the reproductive status of the PPNs and their reproductive efficiency under predation pressure, we examined the impact on egg-laying females (ELF) (**Figures 4B**, **5B**). Similar to gall formation, a substantial reduction in ELF was observed in the treatment pots co-inoculated with predatory nematode at both 6 and 8 weeks (4.5 & 7.5X reduction respectively). In contrast, control pots that only received RKN J2 exhibited a significantly higher number ELF. At week 4, the difference in the number of ELF between control and RKN + P treatments were not statistically significant (*p = 0.2*). Additionally, we investigated the number of J2 present in the root system (**Figures 4C**, **5C**). This metric offers an early indication of how many J2 successfully evaded predation and established themselves within the roots. The assessment of J2 in the root system (**Figure 4C**) further corroborated the discoveries related to gall and egg-laying metrics. Pots co-inoculated with predatory nematodes showed a significant decline in the number of J2 present in the root systems at both 6 and 8 weeks compared to control pots. No significant difference is observed in J2 counts at week 4 between control and RKN + P treatments (*p = 0.8*), suggesting the predatory effects have not yet manifested. By weeks 6 and 8, there is a significant reduction in the number of J2 among the RKN + P treatment when compared to control (*p< 0.001*).

### 3.5 Temporal interaction strength between predatory and root-knot nematodes

**Figure 6:**
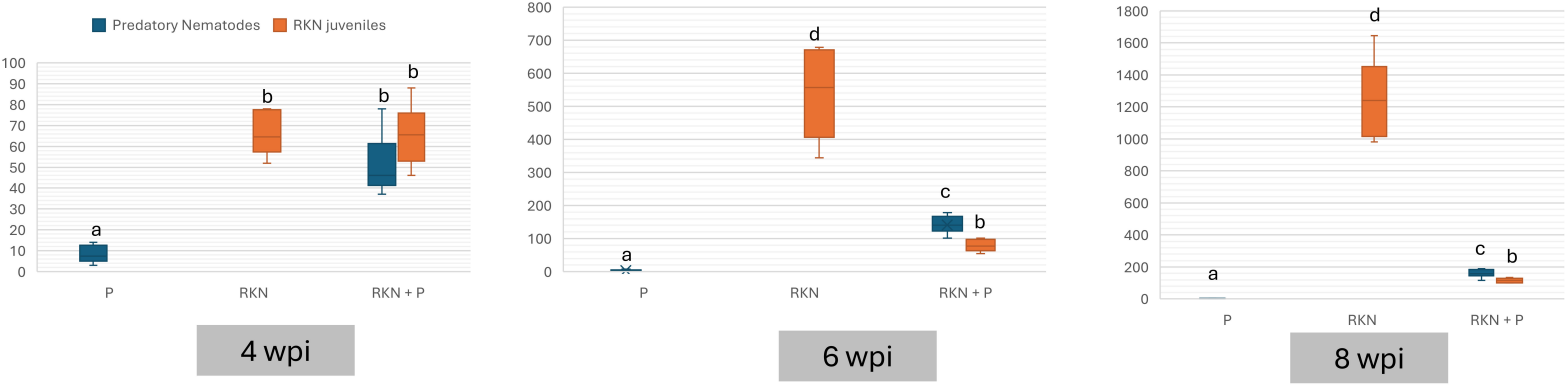
Impact of predatory nematodes on root-knot nematode juveniles across different time points. This figure presents the number of root-knot nematode juveniles (J2) in soil at three key intervals: 4-, 6-, and 8-weeks post-inoculation (wpi) with predator and prey respectively. The data are depicted as box plots showing the distributions for the following treatments: Predatory Nematodes alone (P), Root-Knot Nematodes alone (RKN), and Root-Knot Nematodes with added Predatory Nematodes (RKN + P). Different letters showing statistical differences (*p<* 0.05).

In our previous research, we examined the interactions between predatory nematodes and RKN to understand how predation influences RKN gall formation (**Figure 4A**), disturbance in reproduction (**Figure 4B**) and juvenile (**Figure 4A**) in the root system over time. To further explore these interactions, we explored the number of nematodes in the soil. This experimental setup was designed to evaluate the direct effect of predators on prey populations over different temporal intervals with additional treatment involving predatory nematodes presented as a standalone group. Our data shows no significant differences in the number of RKN juvenile counts between the control groups and predatory and root-knot nematodes (RKN + P) at 4wpi, (**Figure 6**). This observation is supported by the interaction strength (I) values, which hover around 0, and with a mean suppression of 4.9% indicating minimal predatory impact during this period (**Table 2**). At 6 wpi, our data shows a distinct predatory influence, with the RKN + P treatment showing a noticeable decline in RKN juveniles compared to control group (**Figure 6**), which is complemented with the interaction strength (I) (**Table 2**) values range from -0.21 to -0.39, reflecting a top-down effect where predatory nematodes efficiently reduce prey population, evident with high suppression rate. Again, the count of predatory nematodes decreases significantly in the P treatment relative to the RKN + P group, requiring further investigation into the dynamics between predator presence and prey availability. At later time point 8 wpi, the predatory effect becomes most evident (**Figure 6**). The RKN + P group displays the lowest juvenile counts, suggesting a sustained suppression of the RKN population. The interaction strength (I) values, ranging between -4.19 and -4.94, further illustrate the potent and sustained impact of predatory nematodes on reducing RKN numbers. Despite the continuous decline in predatory nematodes in the P treatment, the presence of both predators and prey in RKN + P led to a substantial reduction, highlighting the efficacy of predatory nematode.

**Table 2:** Interaction strength (S) and mean percent suppression (S) of prey in predator-prey relationships.

Samples	4 weeks			6 weeks			8 weeks		
	*I*	*S* %	Mean *S* %	*I*	*S* %	Mean *S* %	*I*	*S* %	Mean *S* %
1	0.00	0	4.9	-0.31	84.9	84.5	-4.59	100.0	100.0
2	0.00	0		-0.39	90.6		-4.32	100.0	
3	-0.13	39.5		-0.36	88.2		-4.94	100.0	
4	0.00	0		-0.30	83.8		-4.44	100.0	
5	0.01	0		-0.21	71.5		-4.30	100.0	
6	0.00	0		-0.29	82.2		-4.53	100.0	
7	0.03	0		-0.34	87.0		-4.84	100.0	
8	0.01	0		-0.35	87.7		-4.19	100.0	

This table summarizes the interaction strength (I) values observed in the predator-prey relationships at 4-, 6-, and 8-weeks post-infestation (wpi). Interaction strength was calculated using formula as described in methods. A total of 8 replicates were employed for each treatment group, and the experiment was conducted three times to ensure reliability and consistency in the results. The data from these replicates were pooled for comprehensive analysis.

## 4 Discussion

In this study, we explored the interactions between banana cultivars and nematode prevalence within Guam’s soil ecosystem, representing a novel investigation into plant-nematode dynamics in this region. For the first time, we characterized the nematode community structure and trophic group dynamics while specifically examining the interplay between predatory nematodes (*Mononchus* spp.) and RKN (*Meloidogyne* spp.). Our results show no differences in the total number of nematodes in the roots across the tested banana cultivars (Blue java, Fiji, Macau, Manila, and Saba) suggesting that nematode infestation levels are either uniformly distributed or that the cultivars exhibit similar susceptibility under the tested conditions. In our study, we identified three genera of plant-parasitic nematodes (PPN) namely, *Helicotylenchus*, *Pratylenchus*, and *Meloidogyne* associated with the tested banana cultivars. Other research has also corroborated our findings, noting the presence of *Meloidogyne*, *Rotylenchulus*, *Helicotylenchus*, and *Pratylenchus* in soil and root samples from banana plants (Mostafa et al., 2021). RKN (*Meloidogyne* spp.) and lesion nematodes (*Pratylenchus* spp.) are recognized among the top ten most important nematodes due to their significant scientific and economic damage (Jones et al., 2013). Both genera are notorious for their detrimental effects on a wide range of crops, leading to substantial yield losses globally (Nicol et al., 2011; Perry and Moens, 2011; Wesemael et al., 2011; Bernard et al., 2017).The recurring dominance of *Meloidogyne* reflects its status as a key pest in numerous agricultural systems, particularly in banana cultivation. This genus’s adaptability and reproductive capacity contribute to its widespread distribution and impact. As highlighted by Van den Bergh et al. (2006); Wesemael et al. (2011); Onkendi et al. (2014), *Meloidogyne* spp. are not only abundant but also capable of causing significant damage to root systems, impairing nutrient and water uptake, which can lead to reduced plant vigor and yield in many crops. Our study is the first to evaluate these specific banana cultivars against nematodes in tropical soil conditions, providing a valuable baseline for future research. Furthermore, in our study, we found an average density of 34 plant-parasitic nematodes per 100 grams of banana roots. This finding suggests a relatively low prevalence of nematodes, particularly when contextualized against established thresholds for nematode damage. Thresholds for nematode damage depend on various factors, including soil type, cultivar, nematode species, and environmental conditions (Blake, 1969; Jones and Milne, 1982; Gowen et al., 2005; Sikora et al., 2018). Literature suggests that densities exceeding 100 nematodes per 100 grams of roots are often identified as critical points where nematodes can begin causing significant stress or damage to banana plants (Seinhorst, 1965; Ferris, 1978; Nicol et al., 2011). Given that our observed density of 34 nematodes per 100 grams of roots falls below the 100 nematodes threshold commonly associated with low damage potential, it indicates suppression of nematode population level in our study may not pose an immediate threat to the health of the banana plants (Trudgill and Phillips, 1997; Moens et al., 2001).This result aligns with findings from previous research which identified thresholds for economic damage in other crops as being in the range of 50 to 100 nematodes per 100 grams of roots (Barker et al., 1994; Barker and Davis, 1996). The findings specific to the Fiji banana cultivar, which showed significantly lower numbers of *Meloidogyne* spp compared to the Manila cultivar. For instance, Davide (1996); De Waele (1996); Speijer and De Waele (1997); Sousa et al. (2024) reported that variations in susceptibility among different banana cultivars often correlate with genetic traits that affect nematode resistance.

This study characterizes, for the first time, the nematode community structure and trophic group dynamics in the context of different banana cultivars in Guam, focusing specifically on the interactions between predatory nematodes (*Mononchus* spp.) and RKN (*Meloidogyne* spp.). Our findings reveal a striking uniformity in total nematode counts across the various banana cultivars, indicating a consistent nematode density in the root-zone soil irrespective of the cultivar. This observation suggests that variations in the genetic makeup of the banana cultivars do not significantly influence different feeding group under the tested conditions. However, it is important to note that plant roots play a crucial role in shaping nematode communities. “In this case, since all tested bananas are of the same species, the similarities in their root systems may limit the influence of cultivar-specific traits on nematode populations. While the current study focuses on a single field using a randomized block design, it is important to consider that environmental or edaphic factors could still play a significant role in shaping nematode communities, as observed in broader studies by Yeates (1999); Zhang et al. (2022). Future research could benefit from exploring these factors across multiple locations to provide a more comprehensive understanding.” The nematode community in our study is characterized by a significant predominance of bacterivores nematodes, constituting approximately 40% of the total population. Their consistent dominance supports previous studies as reported in Pires et al. (2023), highlighting the crucial role of bacterivores nematodes in nutrient cycling and soil health. Recent studies by Schmidt et al. (2020); Fan et al. (2025) also showed that bacterivores nematodes were the most abundant functional group in an organic minimum tillage system, operating as significant markers of soil fertility, increasing crop production. Our findings mirror this trend and emphasize the contribution of bacterivores nematodes in maintaining healthy soil environments conducive to banana cultivation. In contrast to the robust populations of bacterivores, PPNs represented only 13% of the nematode community in our study, indicating effective predation or competitive dynamics with other feeding groups. A recent article published by Wilschut and Geisen (2021); Topalović and Geisen (2023); Li et al. (2024) provides insights into soil nematodes by showing direct and indirect links of both PPNs and free -living nematodes (FLNs) with plant performance. Similarly, in our research the relatively low abundance of PPNs in banana soils may suggest an effective natural regulatory mechanism facilitated by predatory nematodes, which comprised up to 30% of the community. Interestingly, the unexpected prevalence of predatory nematodes within our samples raises important implications for ecological balance in the banana rhizosphere. Unlike our findings, other studies and reviews, such as those by Gowen and Quénéhervé (1990); De Waele and Davide (1998); Brooks (2004); Daneel and De Waele (2017) suggest that plant-parasitic nematodes can often dominate in banana plantations as a consequence of monoculture practices. Overall, our results indicate a substantial proportion of the nematode community comprises beneficial groups like bacterivores and predators, which can suppress PPNs populations and enhance soil health. The observed balance of nematode trophic groups reflects the intricate ecological interactions that underpin successful banana production systems. Our findings support the notion that elevated organic matter levels across the rhizosphere contributed to a favorable environment for beneficial nematodes, thus mitigating the impact of PPNs. In our experiment, the observed high nitrogen and carbon content in the soil may have contributed to the positive influence of nematodes on plant health and development. The interplay between these beneficial nematodes and the nutrient-rich environment likely facilitated improved nutrient cycling and availability, which are essential for optimal plant growth. As nematodes interact with the roots, they can enhance nutrient uptake by increasing root surface area and promoting microbial activity in the rhizosphere, ultimately supporting plant vigor and resilience. These findings align with previous research by Ferris et al. (2004); Wei et al. (2012); Gebremikael et al. (2016), underscoring the importance of maintaining sufficient levels of carbon and nitrogen in soils to maximize the beneficial effects of nematodes. The presence of high concentrations of these nutrients not only creates a favorable habitat for nematodes but also ensures that plants can harness their attributes to enhance growth and crop productivity. In alignment with our studies, research by Timper et al. (2021) demonstrates that plant-parasitic nematode populations are suppressed by predatory nematodes, likely due to the increased organic matter content resulting from winter cover crops, which enhances the biological activity in the soil and contributes to the effective control of root-knot nematodes. In addition, soil microbiomes are known to be associated with subtropical fruit trees that can effectively suppress plant-parasitic nematodes like *M. enterolobii*, with significant reductions in nematode populations correlated to the abundance and diversity of specific microbial strains, as well as soil variables such as organic matter content, pH, and other physicochemical factors (Rashidifard et al., 2022). Further corroborating this, a study by Linford et al. (1938); McBride et al. (2000); Widmer et al. (2002) demonstrated that fields with higher organic matter content experienced reduced incidence of RKN *(Meloidogyne* spp.). Such findings parallel our results, reinforcing the hypothesis that the enhancement of beneficial nematode communities through organic matter amendments leads to improved soil health and pest suppression. However, contrasting reports exist, suggesting that in some agricultural systems, particularly those with organic amendments where organic matter is not well decomposed, certain plant-parasitic nematodes may still thrive. Studies have shown that long-term organic and mineral fertilization significantly influences soil nemato fauna in Sorghum cultivation, with manure application improving soil nutrient content and promoting greater populations of beneficial nematodes, while simultaneously increasing the abundance of PPNs. These findings highlight the importance of targeted soil management practices for optimizing soil health and enhancing crop yields. For example Mcsorley (2011) case studies from Florida demonstrate that while composts and crop residues are commonly used as organic amendments, their role in managing nematode populations was not always effective. Although the application of these amendments frequently improved plant growth and stimulated populations of free-living nematodes, particularly bacterivores, the suppression of PPN was inconsistent. This variability suggests that while organic amendments can enhance overall soil health and plant performance, they do not uniformly guarantee reductions in harmful nematode species which could be explained by dose of organic matter application or level of decomposition in the soil. In discussing the effectiveness of organic amendments in nematode suppression, it is important to note that their impact can vary significantly across different contexts.

Despite these contrasting findings, our study highlights the critical role of organic matter in promoting a balanced nematode community structure which was complemented by our control pot experiments. Utilizing tomato plants as a model system in a controlled pot experiment, we elucidated the complexities of nematode interactions, providing insights into the potential influence of plant cultivars on nematode populations and their associated ecological roles. This study lays the groundwork for understanding the pivotal relationships between plant species and nematodes, which could have significant implications for sustainable agriculture practices in Guam and beyond. The infection (gall severity) in the tomato control pots not only indicate the level of susceptibility of the host plant to nematode infestation but also reflect the nematodes’ ability to manipulate host plant defenses to their advantage using several pathways (Bird and Kaloshian, 2003; Gheysen and Mitchum, 2008; 2011; Nahar et al., 2011; Kyndt et al., 2013; Favery et al., 2016; Gheysen and Mitchum, 2019; Singh et al., 2020). The number of galls formed within the root system serves as a direct measure of nematode impact and the efficiency of their feeding activities. Our controlled pot experiments provided novel insights into the temporal dynamics of predator-prey interactions between *Meloidogyne* spp. and the predatory nematode *Mononchus* spp. The analysis revealed pronounced impacts on gall formation, egg-laying female counts, and juvenile numbers over time, demonstrating that the benefits of predation become more evident over the period of time. This finding emphasizes the potential of predatory nematodes not only to reduce gall formation but also to diminish the reproductive success of plant-parasitic nematodes. A significant reduction in J2 counts in pots co-inoculated with predatory nematodes reflects their predation effectiveness while simultaneously indicating that those J2s that survived are capable of forming galls and continuing the life cycle. This result underscores the cumulative effect of predatory nematodes in controlling juvenile populations over time, suggesting that the impact of predatory nematode on RKN populations becomes more pronounced over time.The concept of temporal dynamics in nematode interactions is novel in the context of nematology and has been relatively underexplored in previous studies. Our findings elaborate on how predatory nematodes impact the life cycle of their prey across different growth stages of both nematodes and host plants. This emphasizes that the timing of biological control strategies is crucial for maximizing their effectiveness and suggests that strategic interventions could be designed to align with the developmental stages of pest populations. This approach contrasts with the predominantly static assessments found in past research, which often lacked temporal analysis, limiting the understanding of these dynamic systems. In our study, we observed clear patterns in the temporal interactions between predator and prey populations, which correspond well with the predictions of the Lotka-Volterra model as reviewed in Stevens (2012). Specifically, predator and prey populations cycle through time as predators reduce the numbers of prey. This reduction in food resources subsequently leads to a decrease in predator abundance and in turn, the diminished predation pressure allows prey populations to rebound. As the predator populations increase, they exert greater strain on the prey populations, acting as a top-down control and pushing them towards a state of decline. As evidenced by our data, incorporating a temporal perspective into nematode management strategies may enhance the control of *Meloidogyne* spp. through natural predation, potentially allowing farmers to optimize interventions based on the developmental stages of both nematode and host plant. Notably, the interaction strength analysis indicated negative values, suggesting that predatory nematodes exert a strong top-down control over their prey, corroborating Lotka-Volterra model. Across our study’s time points, the predatory impact was more pronounced, indicating that intervention strategies focusing on natural predation could lead to more sustainable practices in managing nematode populations. New studies have also found that beneficial entomopathogenic nematodes (EPNs) not only reduce root-knot nematode infestations but also herbivore (*Tuta absoluta*) preferences in tomato plants (Kamali et al., 2022). Nevertheless, research shows that not only do predatory nematodes feed on plant-parasitic nematodes, but other beneficial organisms, such as specific fungi, bacteria, *Serratia marcescens* and *Pseudomonas* also contribute significantly to the control of root-knot nematodes *M.incognita* in tomato plants (Mohamed et al., 2009; Hu et al., 2017; Cetintas et al., 2018; Soliman et al., 2021; Singh and Wesemael, 2022; Ye et al., 2022; Ji et al., 2024; Shi et al., 2025).

In summary, this study provides a detailed assessment of the nematode community dynamics in banana cultivation in tropical soil, highlighting the significant role of beneficial nematodes in mediating plant-parasitic nematode populations. The uniform response of different banana cultivars to nematode infestation underscores the influence of ecological interactions and soil health rather than varietal differences. Our findings advocate for the integration of biological control mechanisms into pest management strategies, emphasizing the potential of predatory nematodes as a potential biopesticide in supporting sustainable agricultural practices. Future research in this area should focus on elucidating the specific interactions between plant roots, compost, and nematode populations to enhance the efficacy of these biological controls in mitigating nematode-induced damage in banana cultivation.

## Data Availability

The original contributions presented in the study are included in the article/**Supplementary Material**. Further inquiries can be directed to the corresponding author.
