# Supplementary material for: The role of predatory nematodes in managing plant-parasitic nematodes: community dynamics and microbial implications in tropical soils
Source: Front Plant Sci. 2025 Dec 11;16:1715934. doi: 10.3389/fpls.2025.1715934 (PMC12738920; doi:10.3389/fpls.2025.1715934)
Supplement: Supplementary file 1 [file DataSheet1.zip › Supplementary Material Presentation 2.pptx]

## Slide 1
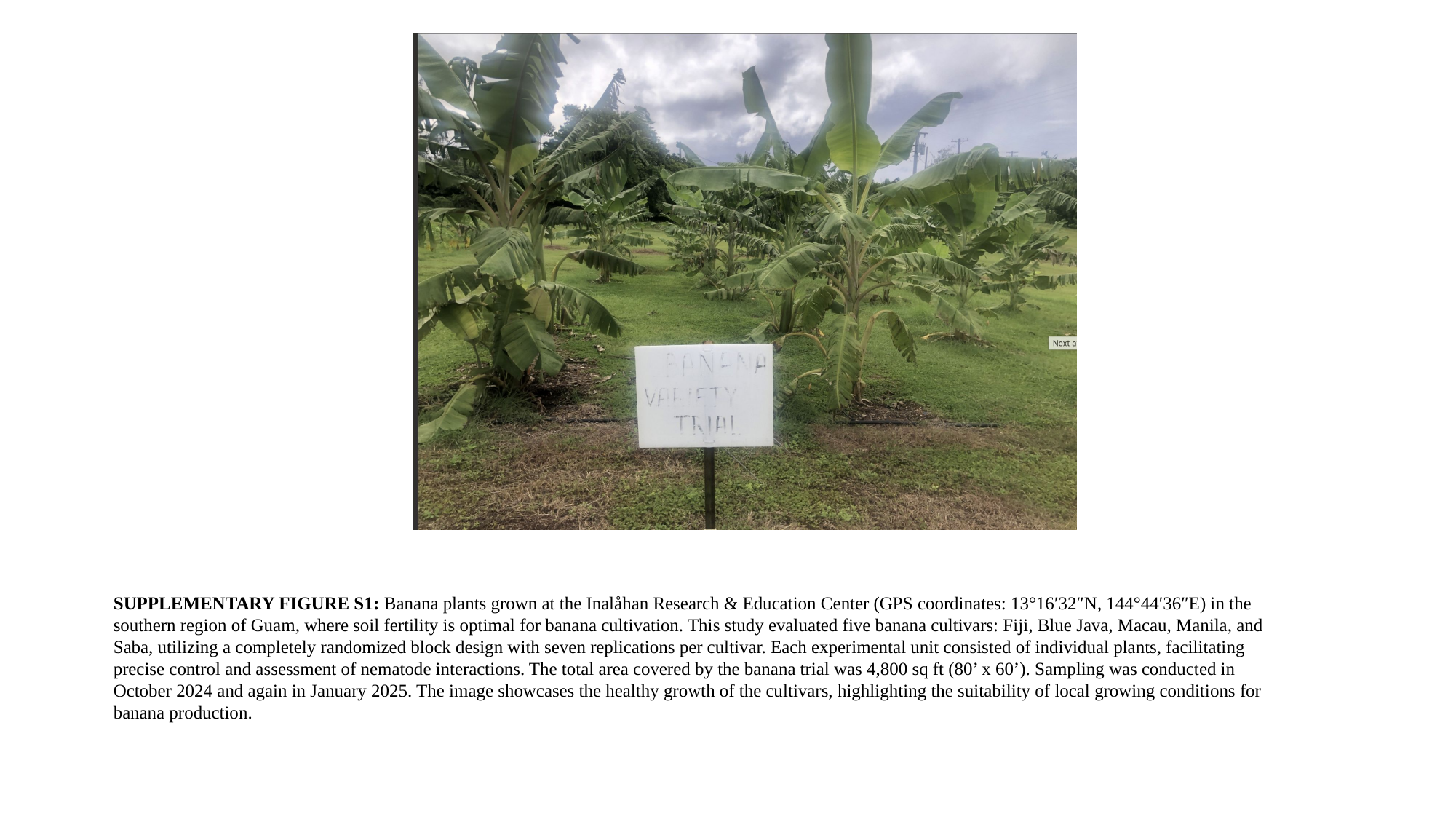

SUPPLEMENTARY FIGURE S1: Banana plants grown at the Inalåhan Research & Education Center (GPS coordinates: 13°16′32″N, 144°44′36″E) in the southern region of Guam, where soil fertility is optimal for banana cultivation. This study evaluated five banana cultivars: Fiji, Blue Java, Macau, Manila, and Saba, utilizing a completely randomized block design with seven replications per cultivar. Each experimental unit consisted of individual plants, facilitating precise control and assessment of nematode interactions. The total area covered by the banana trial was 4,800 sq ft (80’ x 60’). Sampling was conducted in October 2024 and again in January 2025. The image showcases the healthy growth of the cultivars, highlighting the suitability of local growing conditions for banana production.

## Slide 2
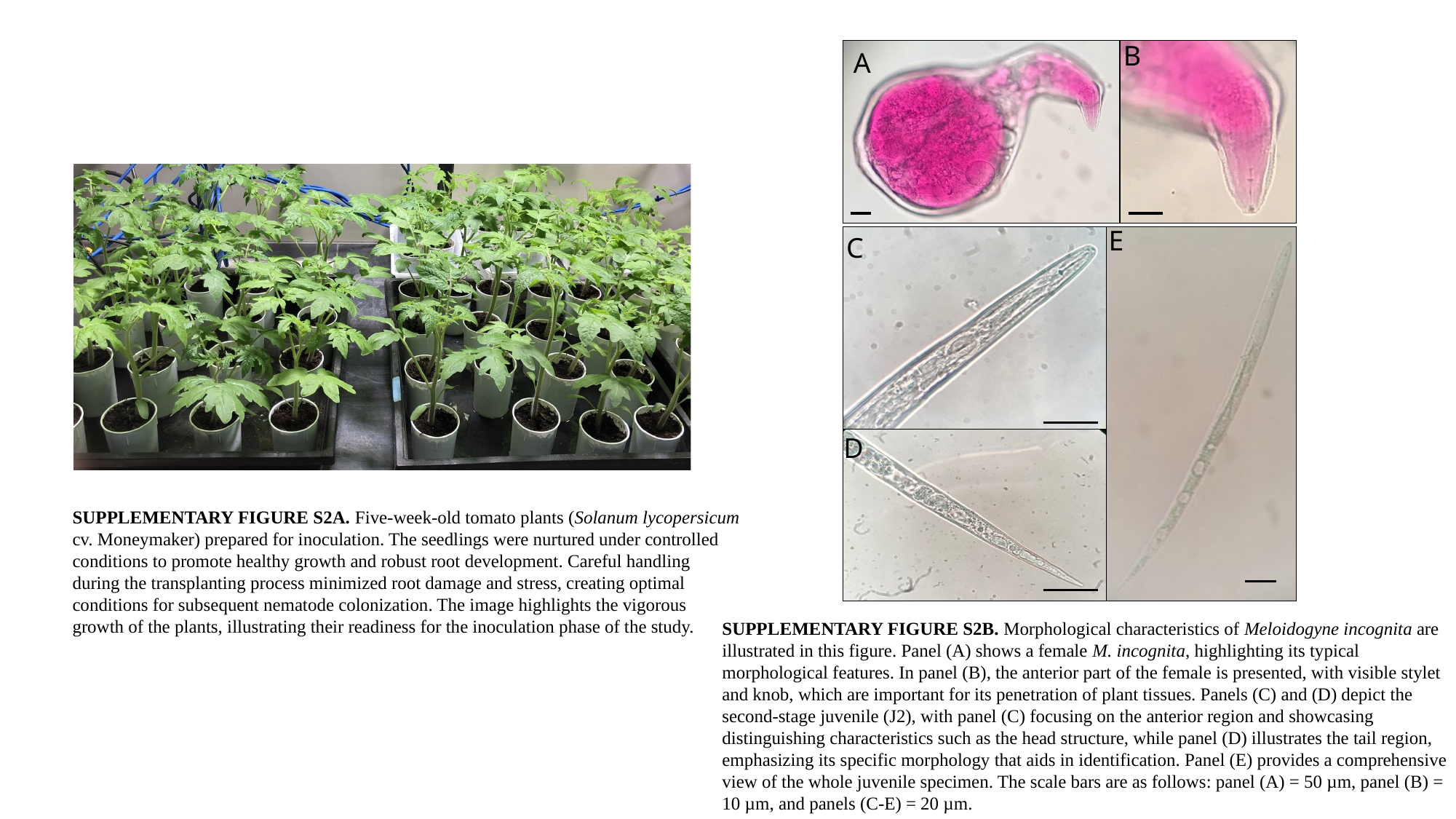

B
A
E
C
D
SUPPLEMENTARY FIGURE S2A. Five-week-old tomato plants (Solanum lycopersicum cv. Moneymaker) prepared for inoculation. The seedlings were nurtured under controlled conditions to promote healthy growth and robust root development. Careful handling during the transplanting process minimized root damage and stress, creating optimal conditions for subsequent nematode colonization. The image highlights the vigorous growth of the plants, illustrating their readiness for the inoculation phase of the study.
SUPPLEMENTARY FIGURE S2B. Morphological characteristics of Meloidogyne incognita are illustrated in this figure. Panel (A) shows a female M. incognita, highlighting its typical morphological features. In panel (B), the anterior part of the female is presented, with visible stylet and knob, which are important for its penetration of plant tissues. Panels (C) and (D) depict the second-stage juvenile (J2), with panel (C) focusing on the anterior region and showcasing distinguishing characteristics such as the head structure, while panel (D) illustrates the tail region, emphasizing its specific morphology that aids in identification. Panel (E) provides a comprehensive view of the whole juvenile specimen. The scale bars are as follows: panel (A) = 50 µm, panel (B) = 10 µm, and panels (C-E) = 20 µm.

## Slide 3
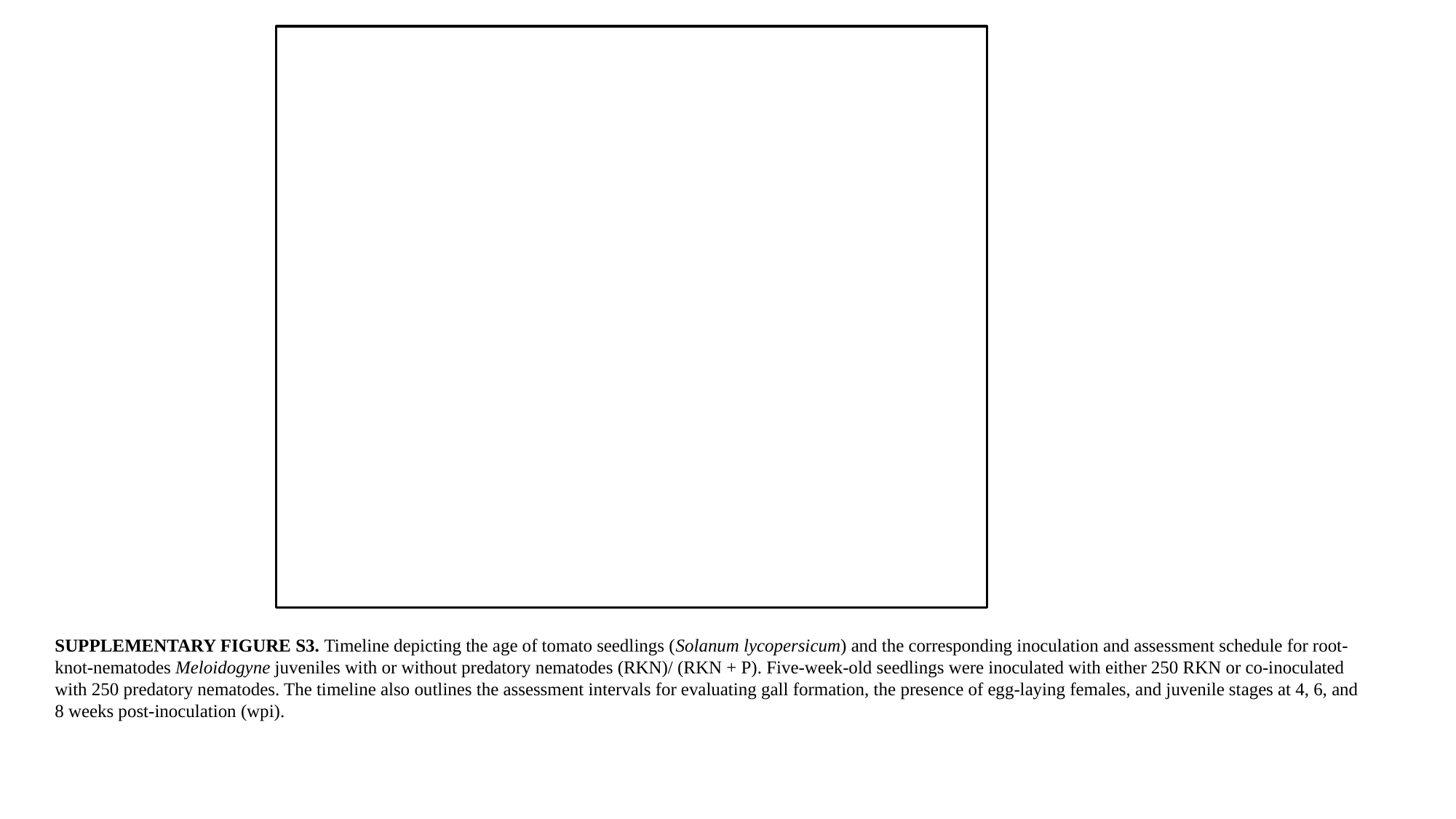

SUPPLEMENTARY FIGURE S3. Timeline depicting the age of tomato seedlings (Solanum lycopersicum) and the corresponding inoculation and assessment schedule for root-knot-nematodes Meloidogyne juveniles with or without predatory nematodes (RKN)/ (RKN + P). Five-week-old seedlings were inoculated with either 250 RKN or co-inoculated with 250 predatory nematodes. The timeline also outlines the assessment intervals for evaluating gall formation, the presence of egg-laying females, and juvenile stages at 4, 6, and 8 weeks post-inoculation (wpi).

## Slide 4
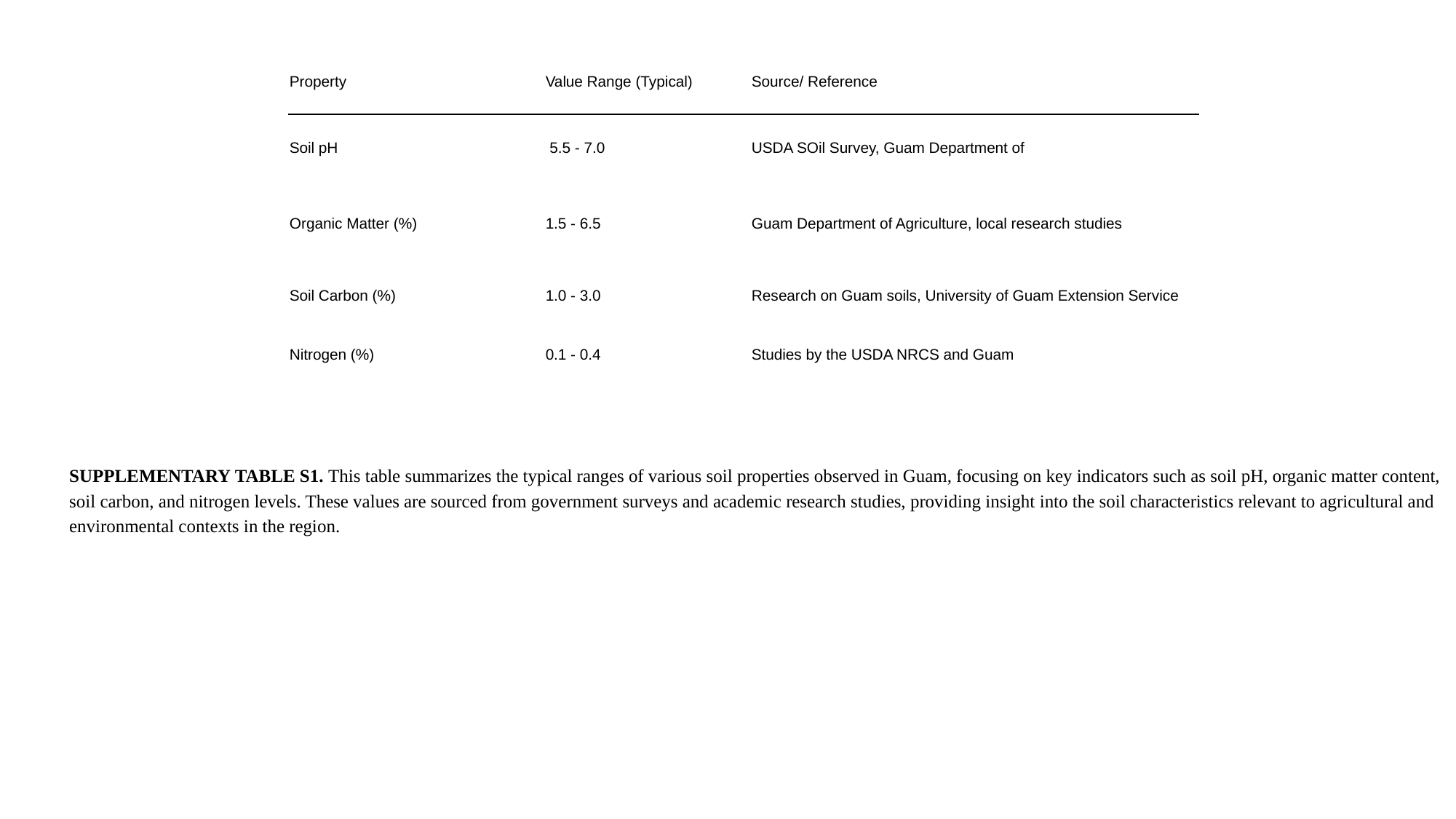

| Property | Value Range (Typical) | Source/ Reference | |
| --- | --- | --- | --- |
| | | | |
| Soil pH | 5.5 - 7.0 | USDA SOil Survey, Guam Department of | |
| | | | |
| Organic Matter (%) | 1.5 - 6.5 | Guam Department of Agriculture, local research studies | |
| | | | |
| Soil Carbon (%) | 1.0 - 3.0 | Research on Guam soils, University of Guam Extension Service | |
| | | | |
| Nitrogen (%) | 0.1 - 0.4 | Studies by the USDA NRCS and Guam | |
SUPPLEMENTARY TABLE S1. This table summarizes the typical ranges of various soil properties observed in Guam, focusing on key indicators such as soil pH, organic matter content, soil carbon, and nitrogen levels. These values are sourced from government surveys and academic research studies, providing insight into the soil characteristics relevant to agricultural and environmental contexts in the region.
